# A retrospective analysis of second-line chemotherapy in patients with advanced gastric cancer

**DOI:** 10.1186/1471-2407-9-110

**Published:** 2009-04-09

**Authors:** Sang Hoon Ji, Do Hyoung Lim, Seong Yoon Yi, Hyo Song Kim, Hyun Jung Jun, Kyoung Ha Kim, Myung Hee Chang, Min Jae Park, Ji Eun Uhm, Jeeyun Lee, Se Hoon Park, Joon Oh Park, Young Suk Park, Ho Yeong Lim, Won Ki Kang

**Affiliations:** 1 Division of Hematology-Oncology, Department of Medicine, Sungkyunkwan University School of Medicine, Samsung Medical Center, 50 Ilwon-dong, Kangnam-gu, Seoul 135-710, Korea

## Abstract

**Background:**

Because treatment of advanced gastric cancer (AGC) patients after failure with first-line chemotherapy remains controversial, we performed this retrospective analysis based on the data obtained from 1455 patients registered in a first-line treatment cohort with respect to receiving or not receiving subsequent chemotherapy.

**Methods:**

The decision for administering second-line chemotherapy was, in most cases, at the discretion of the physician. Seven-hundred twenty-five (50%) received second-line chemotherapy after first-line failure. Univariate and multivariate analyses were performed on the recognized baseline parameters for survival.

**Results:**

At the time of initiating second-line chemotherapy, the patients' median age was 56 years (range, 22 to 86) and 139 (19%) had an Eastern Cooperative Oncology Group (ECOG) performance status of 2 or more. Seven (1%) complete and 108 (15%) partial responses to second-line chemotherapy were observed for an overall response rate of 16% (95% confidence interval [CI], 13 to 19%). The median progression-free and overall survivals, calculated from the start of second-line chemotherapy, were 2.9 months (95% CI, 2.6 to 3.3) and 6.7 months (95% CI, 5.8 to 7.5), respectively. Multivariate analysis revealed that low baseline hemoglobin level (hazard ratio [HR], 0.74; 95% CI 0.61–0.90) and a poor performance status (HR, 0.66; 95% CI, 0.52–0.83) were independent negative prognostic factors for overall survival.

**Conclusion:**

Performance status, along with baseline hemoglobin level, could be used to identify the subgroup of patients most likely to benefit from second-line chemotherapy for AGC.

## Background

Gastric cancer is the most frequently occurring malignancy in Korea, and is one of the main causes of cancer death [1]. For patients with recurrent, metastatic, or advanced gastric cancer (AGC), chemotherapy can improve survival, and possibly, provide significant palliation of symptoms [2,3]. However, over half of patients with AGC who received chemotherapy failed to achieve response and even in these responders, the duration of responses was as short as a few months [4]. Given no standard salvage treatment available in those patients, limited investigation on second-line chemotherapy after first-line failure has been performed [5-7]. The sample size of most studies was small and randomized trial comparing chemotherapy and best supportive care was not yet performed [8].

Despite the lack of evidence for benefit associated with administering salvage chemotherapy, it is a common practice to offer further chemotherapy for AGC patients after first-line failure, because patients and physicians have difficulty with accepting only supportive care without the possibility of systemic anticancer effects. In general, chemotherapy in AGC should be used to prolong survival and improve the quality of life (QOL) of the patients, a factor that is even more important in the case of salvage treatment. Administration of an active and tolerable chemotherapy regimen in a well-selected patient population may have a beneficial effect on QOL, as a direct result of improvements in clinical outcome. Therefore, the knowledge of prognostic and predictive factors, which may contribute to the identification of the subset of patients with a greater likelihood of a benefit from second-line therapy, could be useful. Because well-designed, randomized, controlled clinical trials are lacking in patients with AGC, an exploratory, pooled analysis of small studies or retrospective analysis seems to be an important source of data to allow the definition of optimal treatment, enhance patient counseling, and generate hypotheses for future studies.

In an effort to define the potential role of the second-line chemotherapy in AGC, we performed this retrospective analysis based on the data obtained from 1455 patients registered in our previous retrospective study [9]. The present evaluation was also done with the intent to plan and develop improved therapeutic strategies for AGC patients after first-line failure.

## Methods

We collected follow-up patient data from our cancer registry. We previously performed a retrospective study on 1455 AGC patients who were treated with first-line chemotherapy between September 1994 and February 2005 [9]. All patients had been treated with taxanes- and/or fluoropyrimidine-based combination regimens as their first-line therapy for advanced disease. All the data was prospectively recorded and only the outcome results were updated at the time of analyses. Written informed consent was given by all patients prior to receiving chemotherapy, according to institutional guidelines. This retrospective study was reviewed and approved by the Samsung Medical Center institutional review board (Seoul, Korea).

The decision for administering second-line chemotherapy was, in all cases, at the discretion of the physician. The second-line chemotherapy regimen to be used was determined by the treating physician but, in about half of the patients, in the context of second-line clinical trials. Chemotherapy was repeated every 2–3 weeks according to the regimen. All tumor measurements were assessed after every 2 or 3 courses of chemotherapy, by using abdominopelvic computed tomography (CT) scan and other tests that were used initially to stage the tumor. Tumor response was evaluated and reviewed by an investigator (SHJ) at the time of analysis, according to the Response Evaluation Criteria for Solid Tumors (RECIST) [10].

The primary endpoint of this study was overall survival (OS). The starting point of OS and progression-free survival (PFS) was the first day of second-line chemotherapy. The date of disease progression or death from causes other than AGC was used in calculating PFS. Time to death, whatever the cause, was used to calculate OS. PFS and OS were estimated according to the Kaplan-Meier method and the statistical significance of survival curves between the two groups was tested with a log-rank test. Treatment-free interval was measured from the end of first-line chemotherapy and the start of second-line chemotherapy. To examine the impact of clinical and treatment variables on the outcomes of second-line chemotherapy, multivariate Cox regression models were used. Covariates included were age (below vs. ≥ median), gender, previous gastrectomy, an Eastern Cooperative Oncology Group (ECOG) performance status (0–1 vs. ≥ 2), weight loss (>5%) before treatment, best response to first-line chemotherapy, treatment-free intervals (below vs. ≥ median), number of involved sites (one vs. ≥ 2), metastases (liver, bone, and bone marrow), presence of ascites, baseline chemistry profiles (serum albumin, alkaline phosphatase, and bilirubin), and hemoglobin levels. Laboratory parameters were initially recorded as continuous variables and later dichotomized according to the median value of each variable (below vs. ≥ median). All P values were two-sided, with P < 0.05 indicating statistical significance.

## Results

Patient characteristics are given in Table 1. Seven hundred twenty-five (50%) received second-line chemotherapy after first-line failure. For patients who received supportive care only, reasons for a such decision was available in 609 patients, the main reasons being poor performance status (71%) and patient's refusal (29%). About one-thirds of patients had two or more metastatic disease sites, mostly involving peritoneum and abdominal lymph node. Bone marrow aspiration and biopsy were performed if clinically suspected (n = 69), and a bone marrow involvement was found in 41 patients. Nineteen percent of patients with an ECOG performance status ≥ 2 received second-line chemotherapy. Thirty percent of patients (n = 218) were treated with monotherapy (oral fluoropyrimidines, irinotecan or taxanes), and others received second-line chemotherapy with combination regimens. The most commonly used combination chemotherapy regimen was taxanes/cisplatin (n = 144), followed by the combination of oxaliplatin and leucovorin/fluorouracil (FOLFOX, n = 113), irinotecan-based (n = 109), anthracycline-based (n = 77), and fluoropyrimidine/cisplatin (n = 64). Further third-line chemotherapy was offered to 215 patients (30%) after second-line failure.

**Table 1:** Patient characteristics at the time of second-line chemotherapy

	No. of patients (n = 725)
Median age (range), years	56 (22–86)
Male gender	473 (65%)
Prior therapy	
Surgery	364 (50%)
Adjuvant chemotherapy ± radiotherapy	145 (20%)
First-line chemotherapy*	
FP (± leucovorin) or XP	402 (56%)
ECF or ECX	29 (4%)
Taxane (paclitaxel or docetaxel) + cisplatin	252 (35%)
Irinotecan- or oxaliplatin-based	42 (6%)
Response to first-line chemotherapy	
Responder	143 (20%)
Non-responder or unknown	582 (80%)
Treatment-free interval, months	
Median (range)	2 (1–26)
ECOG^† ^performance status	
0–1	586 (81%)
2 or more	139 (19%)
Involved site(s)	
Two or more involved sites	283 (39%)
Ascites	193
Liver metastasis	139
Bone marrow involvement (n = 69)	41
Baseline laboratory parameters, median	
Hemoglobin, g/l	9.8
Albumin, g/dl	3.4
Alkaline phosphatase, U/l	86
Bilirubin, mg/dl	0.5
Calcium, mg/dl	8.9

*FP, 5-fluorouracil + cisplatin; XP; capecitabine + cisplatin; ECF, epirubicin + cisplatin + 5-fluorouracil; ECX, epirubicin + cisplatin + capecitabine; ^†^ECOG, Eastern Cooperative Oncology Group.

Of a total of 725 patients, 105 could not be evaluated for responses because of the absence of any measurable lesions or early discontinuation of therapy. Objective responses to second-line chemotherapy were noted in 115 patients (response rate, 16%; 95% confidence interval [CI], 13 to 19%) out of which seven were complete responses. Patients who had a poor performance status (≥ 2 in ECOG scale) were significantly less likely to respond to second-line chemotherapy (9% vs. 21%; p = 0.016) compared to those with an ECOG performance status of 0 or 1. Other factors associated with lack of optimal response were the presence of ascites (8% vs. 20%; p = 0.018) and bone marrow involvement (0% vs. 20%; p = 0.038). Response rate was not significantly influenced by age, gender, weight loss, baseline laboratory parameters, bone or liver metastasis, prior response to first-line chemotherapy, or treatment-free interval.

Of the 725 patients included in this analysis, 699 (96%) died. The estimated median PFS was 2.9 months (95% CI, 2.6 to 3.3) and median OS was 6.7 months (95% CI, 5.8 to 7.5). In the univariate model, the estimated OS was significantly shorter for patients with low baseline hemoglobin, ascites, and a poor performance status (Table 2). In the Cox regression model, independent prognostic parameters for OS were performance status and hemoglobin level (Table 3). We also tested whether the survival was modified by interaction between the effect of other significant clinical parameters and performance status; the first-level interaction term between these variables was entered into separate multivariate model. However, the result was not significant. The correlation between performance status and hemoglobin level was not significant (correlation coefficient, 0.048; P = 0.308). This model suggests that AGC patients with low hemoglobin (<9.8 g/l) and poor performance status prior to the initiation of second-line chemotherapy have a risk of death that is two times than that of patients with hemoglobin ≥ 9.8 g/l and an ECOG performance status of 0 or 1.

**Table 2:** Univariate analysis according to baseline clinical parameters

		OS, mo*	95% CI	P
Previous gastrectomy	No	6.7	5.3 to 8.2	.660
	Yes	6.7		
Gender	Male	7.1	5.9 to 8.4	.464
	Female	6.6	5.9 to 7.3	
Age	< median	7.1	5.9 to 8.4	.243
	≥ median	6.5	5.9 to 7.3	
Response to first-line chemotherapy	No	6.4	5.5 to 7.2	.432
	Yes	7.1	5.9 to 8.4	
Treatment-free interval	< median	6.7	5.8 to 7.6	.863
	≥ median	6.8	5.4 to 8.1	
Performance status	0 or 1	7.1	6.2 to 8.1	.001
	≥ 2	5.4	3.8 to 7.1	
Albumin	< median	6.5	5.2 to 7.5	.553
	≥ median	7.1	5.9 to 8.3	
Alkaline phosphatase	< median	6.5	5.7 to 7.4	.886
	≥ median	7.1	5.6 to 8.6	
Bilirubin	< median	6.2	5.3 to 7.1	.766
	≥ median	7.1	5.9 to 8.4	
Calcium	< median	7.2	6.0 to 8.3	.210
	≥ median	5.9	5.1 to 6.8	
Hemoglobin	< median	5.8	5.6 to 6.4	.004
	≥ median	8.1	7.1 to 9.1	
No. of involved site(s)	1	6.7	5.5 to 8.0	.225
	≥ 2	6.4	5.4 to 7.3	
Liver metastasis	No	7.4	5.2 to 9.6	.497
	Yes	6.6	5.8 to 7.5	
Bone metastasis	No	8.4	5.2 to 11.6	.793
	Yes	6.6	5.9 to 7.4	
Ascites	No	7.1	6.2 to 8.1	.038
	Yes	4.7	3.5 to 5.9	
Bone marrow involvement	No	6.7	5.9 to 7.6	.555
	Yes	5.0	2.5 to 7.6	
Weight loss	No	6.7	5.8 to 7.5	.853
	Yes	6.6	3.4 to 9.9	

* OS, median overall survival; 95% CI, 95% confidence interval.

**Table 3:** Multivariate analysis according to baseline clinical parameters

	P	Hazard ratio	95% CI
Presence of ascites	0.704	0.945	0.706 to 1.266
Low hemoglobin	0.002	0.741	0.611 to 0.898
Poor performance status	0.001	0.656	0.516 to 0.834

For exploratory purposes, we compared OS according to second-line chemotherapy regimens given. Of 218 patients who were treated with second-line single-agent therapy, OS was longer, although statistically insignificant, in patients who received irinotecan or taxane (9.3 months and 8.3 months, respectively) than oral fluoropyrimidines (6.2 months; P = 0.245). However, no relevant OS difference between patients who received monotherapy (median, 6.8 months; 95% CI, 5.1 to 8.4) and combination regimens (median, 6.7 months; 95% CI, 5.7 to 7.6) was detected (P = 0.373). On the other hand, OS was significantly longer in patients received third-line chemotherapy (median, 11.5 months; 95% CI, 10.8 to 12.1) than those who were not treated further (median, 4.9 months; 95% CI, 4.5 to 5.4; P < 0.001).

## Discussion

The present analysis of 725 AGC patients who were treated with second-line chemotherapy has demonstrated a strong association between some baseline parameters (i.e., hemoglobin level and a performance status) and OS. The response rate achieved with second-line chemotherapy was 16%. Although this study is retrospective in nature, the results provide a piece of evidence that patients with first-line chemotherapy failure may derive a benefit from second-line chemotherapy.

AGC is an incurable condition where the aim of treatment is to improve survival and to palliate symptoms. Disease may respond to several types of chemotherapy initially, and these treatments have been shown to provide palliation as indicated by improvement in duration and/or quality of survival [2,3]. However, when patients do not respond to chemotherapy or eventually develop disease progression, no established second-line therapy can be offered. There is no evidence that second-line chemotherapy in patients with AGC will result in substantial prolongation of survival and there is potential for toxicity from the treatment. At the time of first-line chemotherapy failure, at least half of the AGC patients are candidates for further treatment. Although it is recognized that there is a declining probability of response with subsequent chemotherapy regimens, favorable outcomes seen in some phase II trials of second-line chemotherapy for AGC[8] In a recent report, we focused on the QOL issues and demonstrated that second-line chemotherapy may be of value [11]. Clearly, second-line chemotherapy may not be beneficial for all patients. It is necessary to better define the sub-population of patients who may benefit from second-line chemotherapy because there is also potential for toxicity and adverse effects from the treatment. Therefore, the identification of factors allowing the selection of patients who are likely to benefit from second-line chemotherapy is an important challenge.

In a pooled analysis of 3 randomized trials, Chau et al [12] investigated the prognostic significance of the baseline factors in 1080 chemotherapy-naïve patients with esophagogastric cancer. They found that poor performance status, metastases to liver and peritoneum, and alkaline phosphatase significantly predicted poor survival. Our previous retrospective study in 1455 AGC patients have revealed that poor prognostic factors were no previous gastrectomy, low albumin, high alkaline phosphatase, bone metastasis, the presence of ascites, and a poor performance status [9].

In the current study, hemoglobin level, as well as performance status, emerged as the significant survival predictor. When interpreting the results, it is of note that this analysis represents only a small sample of patients and half of them had low hemoglobin level at presentation. In this study, we cannot completely exclude the possibility that lower levels of performance status may be reflective of other occult predictors for a poor prognosis. It is possible that sick patients (i.e., patients with a low hemoglobin level and limited survival) may be socially inactive. However, the correlation between performance status and hemoglobin level was not significant.

Low hemoglobin level, in the presence or absence of treatment, is a common finding among AGC patients [13]. A number of factors contribute to the high incidence of cancer-related anemia. These include not only chemotherapy and radiation-induced myelosuppression, but also bleeding, hemolysis, marrow infiltration by tumor, nutritional deficiencies, and cytokine-mediated anemia of chronic disease. The results of the present analysis strongly suggest that patients with poor performance status and/or low hemoglobin level rarely benefited from second-line chemotherapy. It is indicated that second-line chemotherapy in these patients should be given in caution and consideration should be warranted to exclude such patients from future clinical trials.

Besides clinical parameters, appropriate patient selection based on molecular markers is one of the most extensively studied areas in clinical research. While it is still at an early stage and it would take years before we can see clinical application, extensive work is ongoing to identify possible molecular markers, including epidermal growth factor receptor (EGFR) or vascular endothelial growth factor (VEGF) expression [14,15], Her2/neu amplification [16], and excision repair cross-complementing gene 1 (ERCC1) [17], that could be linked to sensitivity or resistance to specific agents, as well as specific genotype variations harbored in different ethnicities.

Although it would be difficult to choose the best second-line chemotherapy regimen from the results of the current study or others, the choice a survival benefit from combination chemotherapy seems unlikely, as there was no significant OS difference between patients who were treated with monotherapy and combination. In salvage setting, the choice of a second-line agent depends on first-line treatment. Because many AGC patients have received first-line chemotherapy with fluoropyrimidine/platinum combination, it seems more prudent to avoid these agents in salvage regimen for reasons of efficacy. Given these considerations, our next step will be to compare single-agent chemotherapy with best supportive care in pretreated AGC patients and to investigate prospectively the relative place of the second-line chemotherapy in the treatment of AGC.

## Conclusion

Selected AGC patients with good performance status should be considered for second-line chemotherapy after first-line failure. Our results show that performance status, along with hemoglobin level, at the time of second-line chemotherapy initiation may provide useful information to predict outcome in AGC patients. With better patient selection, clinical outcomes of AGC patients in second-line setting can be improved. Prospective clinical trials investigating clinical outcomes in association with known prognostic factors such as performance status may be warranted. Furthermore, emerging science and the knowledge of disease may further guide us to develop individualized treatment for AGC patients.

## Competing interests

The authors declare that they have no competing interests.

## Authors' contributions

SHJ drafted the manuscript. DHL, SYY, HSK, HJJ, KHK, MHC, MJP and JEU collected the data and performed the statistical analysis. JL, JOP, YSP, HYL and WKK followed the patients. SHP designed the study and helped with the manuscript. All authors read and approved the final manuscript.

## Pre-publication history

The pre-publication history for this paper can be accessed here:

http://www.biomedcentral.com/1471-2407/9/110/prepub
